# Comparative Efficacy and Safety of Direct Oral Anticoagulants Versus Warfarin in Valvular Atrial Fibrillation: A Systematic Qualitative Review

**DOI:** 10.7759/cureus.86473

**Published:** 2025-06-21

**Authors:** Moiuz Chaudhri, Ahmed D Al Mahrizi, Marc Faltas, Louise Sakowski, Pranesh Rajendran, Jodie Borgmann, Vindhya Rapelli, Zehra Jaffri, Neil Patel, Quang La, Shrujal A Parikh, Frederick Acquah, Christian Kaunzinger, Aditya Mehra

**Affiliations:** 1 Internal Medicine, Ocean University Medical Center, Brick Township, USA; 2 Head of Education, Futures Forward Research Institute, Toms River, USA; 3 Faculty of Medicine and Surgery, University of Malta, Msida, MLT; 4 Osteopathic Medicine, Rowan-Virtua School of Osteopathic Medicine, Stratford, USA; 5 Cardiology, Piedmont Macon Medical Center, Macon, USA; 6 Internal Medicine, Hackensack Meridian School of Medicine, Clifton, USA; 7 Internal Medicine, University of Missouri Kansas City School of Medicine, Kansas City, USA; 8 Internal Medicine, Rowan-Virtua School of Osteopathic Medicine, Stratford, USA; 9 Internal Medicine, Hackensack Meridian Ocean Medical Center, Brick Township, USA; 10 Department of Biology, Texas AM University, College Station, USA; 11 Internal Medicine, Jefferson Einstein Philadelphia Hospital, Philadelphia, USA; 12 Internal Medicine, Hackensack Meridian Ocean University University Medical Center, Brick Township, USA; 13 Cardiology, Hackensack Meridian Ocean University Medical Center, Brick Township, USA

**Keywords:** direct oral anticoagulant therapy, prevention of stroke, subgroup analysis, valvular atrial fibrillation, warfarin therapy

## Abstract

Valvular atrial fibrillation (VAF) increases the risk of thromboembolic events, which require anticoagulant therapy to prevent stroke. Warfarin has become the standard treatment, yet an increasing number of direct oral anticoagulants (DOACs) are becoming popular; however, their role in treating VAF remains unclear. This systematic review evaluated the safety outcomes and effectiveness of DOACs and warfarin treatment for patients with VAF. A systematic database search was performed according to the PRISMA 2020 guidelines through PubMed, Embase, and the Cochrane Library. The study included adult VAF patients who received DOACs or warfarin. Efficacy outcomes were evaluated. The review process included independent screening of studies by multiple authors, who also extracted the data. Due to significant differences in research approaches and outcome measurements, a qualitative analysis was conducted. A total of 537 records were reviewed; three studies met the inclusion criteria. The safety profiles of DOACs matched or surpassed those of warfarin in terms of major bleeding and intracranial hemorrhage occurrence. The efficacy results showed comparable outcomes, but researchers observed distinct results on the basis of valve type. Although existing evidence does not support the use of DOACs in patients with mechanical valves or severe mitral stenosis, our results suggest that they may be safe and effective alternatives to warfarin. Due to the limited availability of high-quality data, further randomized controlled trials are needed to develop evidence-based anticoagulation strategies.

## Introduction and background

Atrial fibrillation is a major public health concern that has led to considerable health problems and death rates because it affected approximately 33.31 million people worldwide in 2019 [1]. Some patients with atrial fibrillation (AF) also have valvular heart disease (VHD), which is generally associated with increased thromboembolic risk [2]. Although there is widespread use of vitamin K antagonists (VKAs), such as warfarin, for stroke prevention, some challenges exist, such as dietary restrictions and regular blood, to monitor therapeutic levels [3].

Atrial fibrillation continues to increase as a global health crisis because deaths due to this condition increased from 117,000 in 1990 to 315,000 in 2019, representing a 169.2% increase [4]. The management of AF is challenging in patients with VHD because these conditions include different types of valvular disease, such as aortic stenosis and mitral regurgitation, and result in different levels of thromboembolic and bleeding risks [5]. VKAs such as warfarin present some limitations, including the requirement for routine monitoring and dietary restrictions [6,7]. Coagulation cascade mechanisms of various anticoagulants were identified, including the direct oral anticoagulants (DOACs) apixaban, rivaroxaban, and dabigatran, along with the traditional anticoagulant warfarin and their primary targets in the common pathway [7].

## Review

Methods

Protocol Registration

This systematic review was completed using the Preferred Reporting Items for Systematic Reviews and Meta-analyses (guidelines) [8]. The protocol was registered on PROSPERO (CRD420250653296).

Eligibility Criteria

Studies were eligible for inclusion if they contained primary data comparing direct oral anticoagulants (DOACs) to warfarin in adult patients diagnosed with valvular atrial fibrillation (VAF). The included studies reported at least one of the following outcomes: ischemic stroke, systemic embolism, major bleeding, or all-cause mortality. Both randomized controlled trials (RCTs) and observational cohort studies were considered. Studies were excluded if they lacked a warfarin comparator arm, did not report the predefined outcomes, or lacked full-text access. Case reports, editorials, letters, commentaries, and systematic reviews were also excluded from the analysis.

Information Sources and Search Strategy

This review utilized four major databases for article retrieval: PubMed, Embase, and Cochrane. The initial database search was performed in February 2025. A single unified search string was applied across all platforms to ensure consistency. The string was constructed by identifying appropriate MeSH terms and commonly used terminology connected via Boolean operators. The final search string was ("valvular atrial fibrillation" OR "valvular AF") AND ("direct oral anticoagulants" OR "DOAC" OR "dabigatran" OR "apixaban" OR "rivaroxaban" OR "edoxaban") AND ("warfarin" OR "vitamin K antagonist") AND ("stroke" OR "ischemic stroke" OR "bleeding" OR "mortality"). In addition to database queries, manual searches of references were done to ensure a comprehensive search strategy. Retrieved citations were imported into EndNote for management, and duplicate entries were removed. The articles were then uploaded to Rayyan.ai for screening, where two independent reviewers conducted a manual review to determine eligibility. No database-specific syntax adjustments were needed.

Study Selection Process

**Figure 1:**
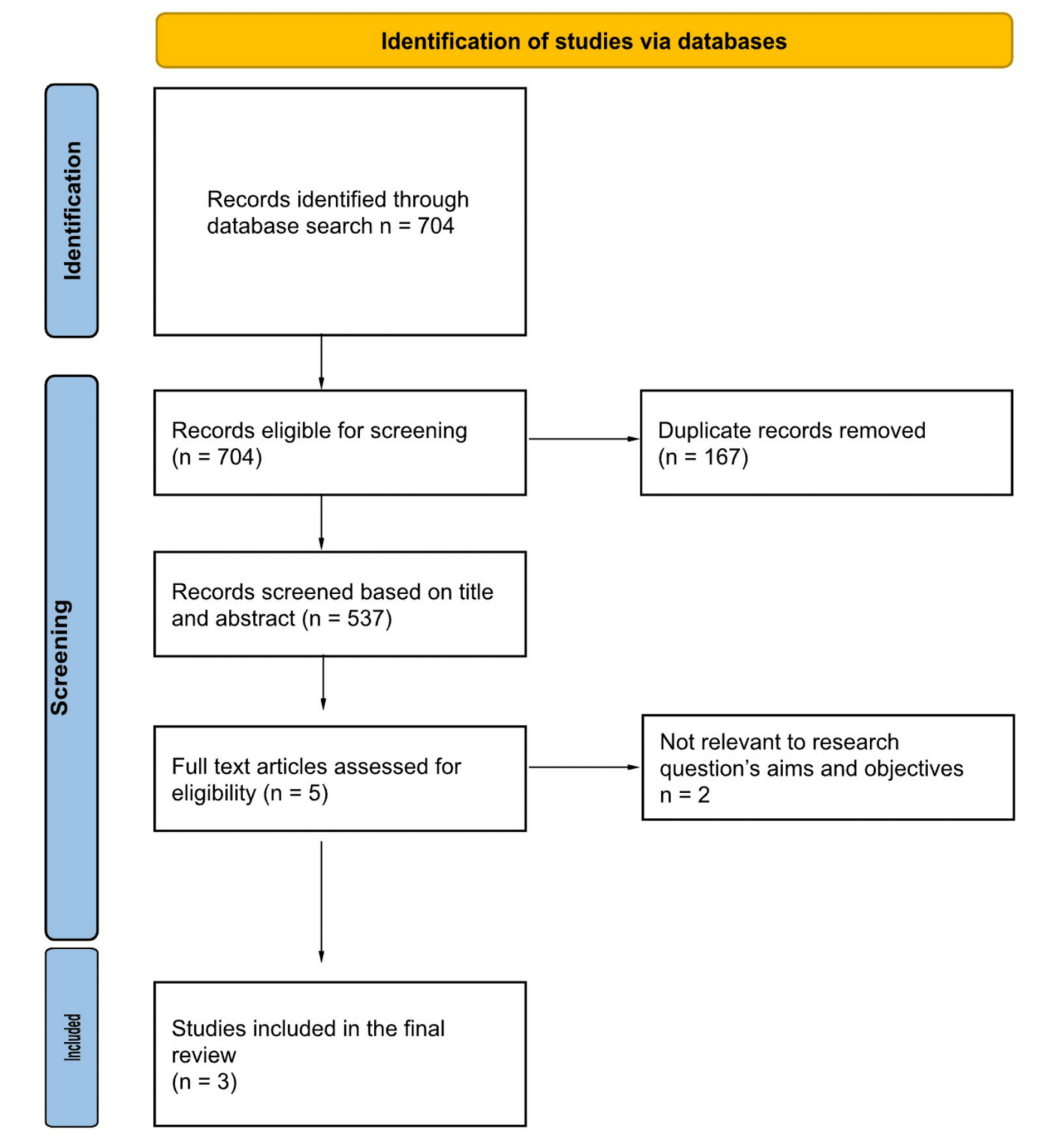
PRISMA flow diagram of the study selection process

Following duplicate detection, each article was screened by title and abstract to determine its relevance on the basis of the eligibility criteria. Full-text reviews were conducted on articles that met the initial screening thresholds. The screening was independently conducted by two reviewers (MC and AM). Any disagreements were resolved by a third reviewer (ZR), who also provided oversight to ensure consistency and minimize selection bias. For articles without accessible full text, the corresponding authors were contacted three times over a four-week period. If no response was received, the article was excluded (Figure 1).

Relevant articles that reported extractable statistical data were included, and the data were systematically extracted into a standardized data collection sheet. The variables extracted included the following: first author, publication year, country, study design, sample size, patient demographics, type of valvular pathology (such as mitral repair or bioprosthetic valve), type of anticoagulant used, duration of follow-up, hazard ratios (HRs) with corresponding 95% confidence intervals for ischemic stroke, major bleeding, and all-cause mortality. The extracted data were collated and analyzed via R software (version 4.3.0).

Risk of Bias Assessment

The risk of bias for each study was assessed via the ROBINS-I (Risk of Bias in Nonrandomized Studies of Interventions) tool [9]. Each domain was graded as having a low, moderate, or serious risk of bias. The results of the bias assessment were visualized via ROBINS, which produced both a traffic light plot and a domain summary bar plot. Most studies demonstrated a moderate risk of bias, although the findings of Mentias et al. were rated as serious because of concerns regarding confounding control and inconsistent outcome definitions [10].

Data Synthesis and Statistical Analysis

A systematic review was conducted via a random-effects model (DerSimonian and Laird method) to account for between-study variability. Pooled HRs and 95% confidence intervals (CIs) were calculated to compare DOACs to warfarin in terms of ischemic stroke risk. Fixed effects models were also generated to evaluate the consistency of the results. Heterogeneity was assessed via the I² statistic, with a value above 50% indicating substantial heterogeneity. Additional metrics included Tau² and the Q statistic. For the pooled ischemic stroke analysis, the I² value was 95.6% (95% CI: 91.5%-97.7%), the Tau² value was 0.0927 (95% CI: 0.0229-1.2682), and the Q statistic was 67.50, with a p-value < 0.0001. Subgroup analyses were conducted for different valve subtypes (mitral repair versus bioprosthetic).

Quality Assessment and Publication Bias

**Figure 2:**
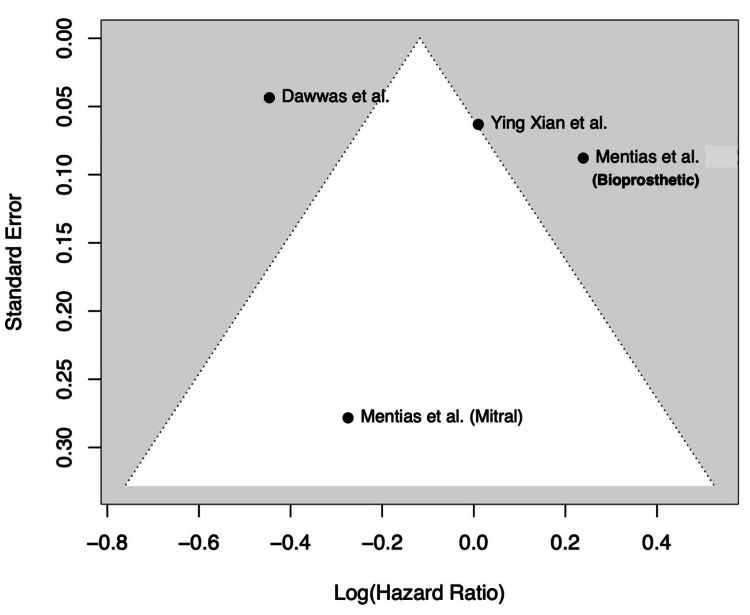
Funnel plot of hazard ratios for ischemic stroke References [10-12]

Although a formal GRADE (Grading of Recommendations Assessment, Development and Evaluation) assessment of certainty was not performed owing to the limited number of studies and the observational nature of most data, a qualitative assessment of evidence strength was conducted. This included evaluation of potential risk of bias, inconsistency among results, indirectness of evidence, imprecision of effect estimates, and potential publication bias. Funnel plots were used to visually assess asymmetry. Despite the small number of included studies, no major evidence of publication bias was identified (Figure 2).

Study Selection

Among the 537 studies deemed eligible after initial screening, three studies subsequently met the inclusion criteria for comprehensive review and final analysis (Figure 1). These were both RCTs and retrospective cohort studies. Taken together, all the selected studies compared DOACs to warfarin in VAF patients. The literature was also carefully selected according to pre-specified eligibility criteria that focused on assessing both the efficacy and safety of DOACs for their ability to prevent ischemic stroke in this patient population.

Study characteristics

The four studies included in the analysis had very different characteristics, especially in terms of the patient populations involved, the types of heart valves affected, and definitions of clinical outcomes. The studies included a mix of adult patients with VAF. Many of the study populations suffered from comorbidities such as hypertension and diabetes. The main endpoint across all four studies was ischemic stroke, with the secondary endpoint being major bleeding events (including intracranial hemorrhage). The follow-up in the studies varied from a matter of months to longer-term outcomes of months to even years (Table 1).

**Table 1:** Baseline characteristics of the included studies DOAC: direct oral anticoagulants; MACE: major adverse cardiac events

Author	Year	Country	Type of Study	Sample Size (n)	Age (Mean ± SD)	Male (%)	Hypertension (%)	Smoking (%)	Diabetes (%)	Intervention Group/Type	Control Group	Group Outcome Measures	Key Outcomes
Mentias et al. [10]	2022	USA	Cohort Study	3,093 DOACs, 4,996 Warfarin	78.0 ± 8.1 DOACs, 76.4 ± 8.1 Warfarin	42.4 DOACs, 44.6 Warfarin	93 DOACs, 90.3 Warfarin	7.1 DOACs, 6.3 Warfarin	40.9 DOACs, 36.8 Warfarin	DOACs (Apixaban, Rivaroxaban, Dabigatran)	Warfarin	Mortality, Stroke, Bleeding	DOACs associated with lower risk of mortality and major bleeding
Dawwas et al. [11]	2021	USA	Cohort Study	46,276 DOACs, 34,967 Warfarin	75.3 ± 10.6 DOACs, 81.4 ± 9.7 Warfarin	55.1 DOACs, 55.2 Warfarin	87.6 DOACs, 85.0 Warfarin	10.4 DOACs, 8.8 Warfarin	34.0 DOACs, 34.3 Warfarin	DOACs (Apixaban, Dabigatran, Rivaroxaban)	Warfarin	Stroke, Bleeding, Mortality	DOACs associated with lower risk of ischemic stroke and major bleeding
Xian et al. [12]	2019	USA	Cohort Study	4,041 DOACs, 7,621 Warfarin	80 (74-86) Median	56.3 DOACs, 56.3 Warfarin	81.3 DOACs, 80.9 Warfarin	N/A	25.0 DOACs, 26.2 Warfarin	DOACs (Apixaban, Dabigatran, Rivaroxaban)	Warfarin	Stroke, Mortality, Major Bleeding	Improved outcomes with DOACs in home time, mortality, and MACE

Meta-Analysis Results

The purpose of this meta-analysis was to compare the efficacy of DOACs and warfarin in preventing ischemic stroke in patients with valvular atrial fibrillation (VAF). HRs for each study were computed. Both the common and random-effects models were employed to pool the results. HRs for ischemic stroke were extracted from all included studies and summarized in a forest plot.

**Figure 3:**
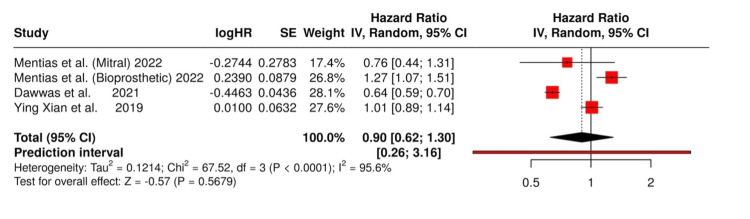
Forest plot of hazard ratios for ischemic stroke References [10-12]

Mentias et al. reported an HR of 0.76 (95% CI: 0.44-1.31) in a mitral valve repair cohort and an HR of 1.27 (95% CI: 1.07-1.51) in a bioprosthetic valve cohort [10]. Dawwas et al. reported an HR of 0.64 (95% CI: 0.59-0.70), and Xian et al. reported an HR of 1.01 (95% CI: 0.89-1.14) [11,12]. These results show varying outcomes across studies and subgroups, with differences in effect sizes and statistical significance (Figure 3).

The common effect (fixed-effect) model showed a pooled HR of 0.80 with a 95% CI of 0.75-0.85 (p < 0.0001), suggesting that DOACs are more effective than warfarin in reducing the risk of ischemic stroke. The random-effects model, which takes heterogeneity into account, produced a pooled HR of 0.90 (95% CI: 0.55-1.48) and a p-value of 0.5524, indicating that there was no significant difference when the two anticoagulants were matched across studies. This difference in results means that while with DOACs, there may be a slight advantage for stroke prevention under the common effect model, any benefit disappears when heterogeneity is factored in.

The heterogeneity analysis revealed a high degree of variability across studies, with an I2 statistic of 95.6% (95% CI: 91.5%-97.7%), indicating substantial inconsistency from one study to the next. This phenomenon occurs in studies where identical patients are often not represented. The Tau² value, which was barely significant at 0.0927 (95% CI: 0.0229-1.2682), and the Q statistic (Q = 67.50, df = 3, p < 0.0001) further confirmed significant heterogeneity.

Publication Bias

Figure 2 shows that there was no evidence of publication bias, with studies symmetrically distributed around the pooled effect estimate.

Risk of Bias

**Figure 4:**
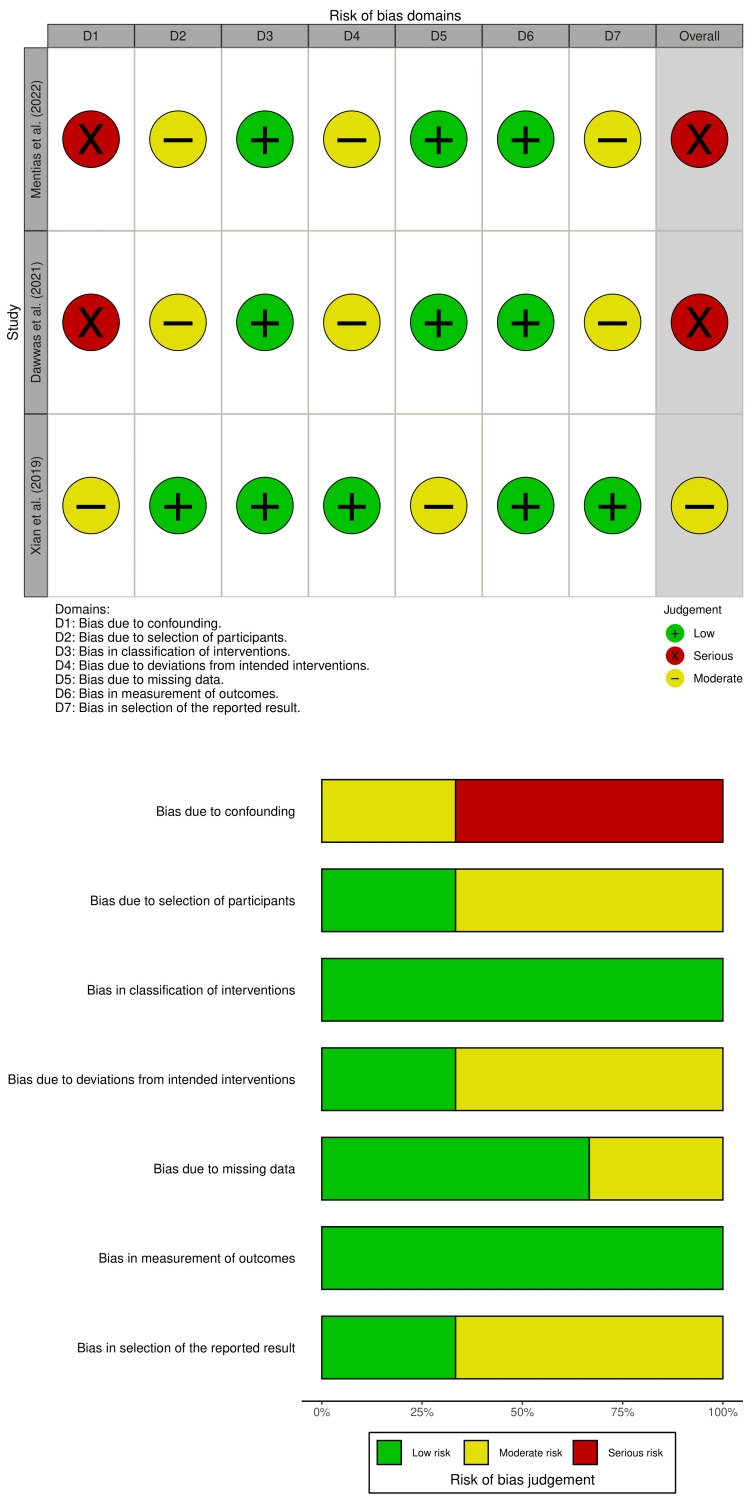
Risk of bias assessment via the ROBINS-I for the included studies

The ROBINS-I tool was used to evaluate the risk of bias in the studies reviewed through this assessment, which revealed that different studies exhibited various levels of bias in their research. The traffic light plot (Figure 4) indicated that most studies presented serious risks across multiple essential domains: multiple studies failed to control for confounding variables, which might have influenced their research outcomes. The selection of participants became biased because different studies used different criteria to include or exclude participants. Multiple studies contained missing follow-up data, which were not adequately managed, thus potentially altering their research findings. The measurement of outcomes revealed inconsistent methods for subject accounting and parameter measurement, especially concerning bleeding events and adverse reactions. Most studies received a moderate risk of bias assessment, but Mentias et al. reported a higher risk of bias [10].

Quality Assessment

A traffic light plot (Figure 4) was used to evaluate the quality of the studies by assessing risk levels as low, moderate, and serious; the results revealed that some studies had no bias, but others had increased levels of concern because of outcome measurement data and incomplete data.

The studies had a limited scope but maintained an acceptable level of quality. The available data enabled us to evaluate the effectiveness and safety of DOACs for preventing ischemic stroke in patients with atrial fibrillation. The studies contained significant heterogeneity and biases, which reduced the reliability of the combined results (Figure 4).

Discussion

This systematic review and meta-analysis of three studies comparing DOACs to warfarin in patients with VAF evaluated the safety and efficacy of various anticoagulation regimens [10-12]. The principal outcome was ischemic stroke, whereas the secondary events were significant bleeding and mortality. The evidence indicates that DOACs are either equivalent to or more effective than warfarin in terms of safety and efficacy, especially in minimizing bleeding-related problems. These findings, albeit constrained by research heterogeneity and patient variability, endorse the utilization of DOACs in suitably selected VAF patients.

The results of individual studies show that DOAC effectiveness varies substantially between patients who fall under different VAF categories. A study by Mentias et al. demonstrated that DOACs reduced the risk of ischemic stroke by 24% in patients who received mitral valve replacement (HR: 0.76) [10]. The results were not statistically significant. The warfarin group presented a notably greater stroke risk in the bioprosthetic valve cohort, which made DOACs the preferred treatment option (HR: 1.27). Dawwas et al. reported that DOACs reduced stroke risk by 36% (HR: 0.64)[11]. However, Xian et al. reported no difference between the two anticoagulants (HR: 1.01) [12]. This research indicates that additional studies are needed to conduct a comprehensive evaluation of this population and to determine how patient characteristics and valve types affect treatment outcomes.

This study on DOACs and warfarin in VAF patients demonstrated that DOACs delivered superior results to warfarin in terms of both safety and effectiveness. Research has shown that DOACs decrease the risk of ischemic strokes and minimize major bleeding risks better than warfarin [11].

Research conducted by Martha et al. revealed that DOACs were more effective than warfarin for VAF patients by reducing stroke or systemic embolism by 24% (HR: 0.76; 95% CI: 0.67-0.87) and intracranial hemorrhage by 58% (HR: 0.42; 95% CI: 0.22-0.80)[13]. A study by Bitar et al. demonstrated that DOACs offer superior protection against stroke or systemic embolism (RR: 0.80; 95% CI: 0.68-0.94) and intracranial hemorrhage (RR: 0.40; 95% CI: 0.24-0.66) [2]. These studies indicate that DOACs offer superior therapeutic benefits for most patients with VAF [2,13].

In order to maximize the benefit of DOACs, their use should be tailored to individual patients with VAF. Selection must consider valve type, coexisting medical conditions, and overall risk profile. Existing literature suggests that DOACs provide treatment outcomes comparable to warfarin in patients with bioprosthetic valves, thus providing a practical alternative by eliminating regular international normalized ratio (INR) monitoring and frequent blood draws [10,14]. Warfarin remains the standard treatment for patients with high thromboembolic risk because it provides both safety and effectiveness. The RE-ALIGN trial established that dabigatran treatment resulted in greater thromboembolic complications and bleeding events than warfarin treatment did for patients with mechanical heart valves [15].

The study showed significant statistical heterogeneity (I² = 95.6%) due to differences between-study subjects, between valve types and anticoagulation methods, and outcome measurement protocols. The generalization of findings to specific population groups becomes less possible because of heterogeneity, which makes aggregated statistics less meaningful. The general trends from multiple studies indicating DOAC superiority should not be disregarded, even though they demonstrate limited application to particular subgroups [10-13].

It is important to note that a couple of landmark trials were not included in our review due to eligibility criteria. Specifically, the ENGAGE AF-TIMI 48 (edoxaban in AF) trial was left out because it enrolled patients with nonvalvular AF [16]. Since this study excluded patients with moderate-to-severe MS or mechanical heart valves, the results also do not apply to the VAF population addressed in our analysis. Incorporation of such studies would result in clinical heterogeneity and reduce the specificity of this analysis. In addition, the RE-ALIGN study, which specifically enrolled patients with mechanical heart valves to assess the effects of dabigatran, was stopped early on the basis of safety, because thromboembolism and bleeding events were higher in the DOAC arm [15]. The trial focused on a sub-cohort of the VAF population, but the early termination of the trial and not aligning with our objectives led to being excluded.

Limitations

This systematic review and meta-analysis have various limitations in their design. The small number of included studies and the high heterogeneity among them limit the generalizability of our findings. The majority of the included studies used observational cohort designs instead of RCTs, which makes them more susceptible to bias from confounding variables and selection effects. The GRADE criteria rate the overall quality of evidence as being between low and moderate levels.

The studies included in this review used different definitions of VAF, anticoagulant dosing protocols, and clinical endpoints such as stroke and bleeding, which might affect the outcome comparison. Most studies failed to present anticoagulation initiation timing-based outcome results, and they did not fully analyze bleeding and stroke events by specific valve types.

Several studies have used administrative claims data, which has resulted in inconsistent reporting of essential clinical variables, including warfarin INR, renal function, and DOAC regimen adherence. The limited number of studies makes it impossible to eliminate publication bias completely, and no research has evaluated outcomes extending past their observed follow-up durations.

## Conclusions

This review provides an updated analysis of existing evidence that compares DOACs to warfarin in VAF. The evidence shows that DOACs match warfarin in terms of efficacy and safety but yield better results in certain patient groups who have mitral valve repair or bioprosthetic valves; however, the evidence is limited by diverse study designs, inconsistent outcomes, and insufficient RCTs. The results indicate that DOACs could serve as an appropriate choice for particular VAF patient groups, but healthcare providers must remain vigilant because these applications exist outside approved indications. Additional high-quality prospective studies must be conducted to determine the specific role of DOACs in different valvular subtypes and develop future guideline recommendations.
